# Depression, antidepressant use, and the risk of type 2 diabetes: a nationally representative cohort study

**DOI:** 10.3389/fpsyt.2023.1275984

**Published:** 2023-12-06

**Authors:** Hyewon Kim, You-Bin Lee, Jungkuk Lee, Dongwoo Kang, Gyuri Kim, Sang-Man Jin, Jae Hyeon Kim, Kyu Yeon Hur, Hong Jin Jeon

**Affiliations:** ^1^Department of Psychiatry, Depression Center, Samsung Medical Center, Sungkyunkwan University School of Medicine, Seoul, Republic of Korea; ^2^Division of Endocrinology and Metabolism, Department of Medicine, Samsung Medical Center, Sungkyunkwan University School of Medicine, Seoul, Republic of Korea; ^3^Data Science Team, Hanmi Pharm. Co., Ltd., Seoul, Republic of Korea; ^4^Department of Health Sciences and Technology, Department of Medical Device Management and Research, and Department of Clinical Research Design and Evaluation, Samsung Advanced Institute for Health Sciences and Technology (SAIHST), Sungkyunkwan University, Seoul, Republic of Korea

**Keywords:** depression, antidepressants, type 2 diabetes, diabetes mellitus, risk factors

## Abstract

**Background:**

Previous studies have reported that depression can increase the risk of type 2 diabetes. However, they did not sufficiently consider antidepressants or comorbidity.

**Methods:**

The National Health Insurance Sharing Service database was used. Among the sample population, 276,048 subjects who had been diagnosed with depression and prescribed antidepressants (DEP with antidepressants group) and 79,119 subjects who had been diagnosed with depression but not prescribed antidepressants (DEP without antidepressants group) were found to be eligible for this study. Healthy controls (HCs) were 1:1 matched with the DEP with antidepressants group for age and sex. We followed up with them for the occurrence of type 2 diabetes.

**Results:**

In the group of DEP with antidepressants, although the risk of type 2 diabetes increased compared to HCs in a crude analysis, it decreased when comorbidity was adjusted for. In the group of DEP without antidepressants, the risk of type 2 diabetes decreased both in the crude model and the adjusted models. The risk varied by age group and classes or ingredients of antidepressants, with young adult patients showing an increased risk even in the fully adjusted model.

**Conclusion:**

Overall, those with depression had a reduced risk of type 2 diabetes. However, the risk varied according to the age at onset, comorbidity, and type of antidepressants.

## Introduction

Depression is a common mental disease with a lifetime prevalence of 7.7% in South Korea (1) and a global lifetime risk of ~1 in 6 individuals (2). It is associated with a significant morbidity (3). When a patient is diagnosed with depression, he or she is recommended to undergo pharmacotherapy (mostly, antidepressants), psychotherapy, or combined treatment (4). Even after a depressive episode is remitted through a successful treatment, many patients relapse or go through a chronic course. Type 2 diabetes is also a prevalent disease. Globally, 537 million people were estimated to have diabetes in 2021, with type 2 diabetes accounting for more than 90% of diabetes (5). High morbidity and mortality associated with microvascular and macrovascular complications in type 2 diabetes can cause great distress to patients and caregivers with a large economic burden (6, 7).

Previous studies have suggested that depression might be a risk factor for type 2 diabetes. The pooled relative risk of incident type 2 diabetes in people with depression ranges from 1.18 to 1.60 (8–12). However, since antidepressant medications are commonly prescribed in patients with depression, they could mediate the relationship between depression and the subsequent occurrence of type 2 diabetes. A recent meta-analysis has reported that the use of antidepressants is generally associated with an increased risk of type 2 diabetes with a pooled hazard ratio of 1.24 (13). However, previous studies have limitations such as not limiting study participants to patients with depression (14–16), enrolling only a specific age group (17–20), relying on self-report for diagnosis of diabetes (19), not considering comorbid physical diseases (21–23), or not considering the type of antidepressants (18, 20–22, 24).

Considering the high prevalence and morbidity of depression and type 2 diabetes in addition to their high correlation, identifying risk factors for type 2 diabetes in patients with depression can be an important strategy for screening and early detection of type 2 diabetes. However, since psychiatric medications (particularly, antidepressants) and several physical conditions could affect the relationship between depression and type 2 diabetes, an analysis considering these factors is crucial.

Thus, the objective of this study was to examine the association between depression, antidepressant use, and the occurrence of type 2 diabetes considering its potential risk factors using a nationally representative population.

## Methods

### Study design and data source

This was a retrospective cohort study using data from the National Health Insurance Sharing Service (NHISS) database of the National Health Insurance Service (NHIS) of South Korea (25, 26). NHIS is a public organization responsible for operating a mandatory universal health insurance program. It covers nearly 97% of the total South Korean population. The remaining 3% is covered by the Medical Aid Program. The NHISS database includes data of medical services and claims such as inpatient, outpatient, emergency room visits, pharmacy data, and health screening programs.

NHISS data are anonymized. The Institutional Review Board of Samsung Medical Center exempted this study from review as it involved retrospective analyses of de-identified data (no. SMC 2019-09-030).

### Case identification

Data of a sampling population of major psychiatric disorders extracted from the NHIS database were used. The sample population included those initially diagnosed with psychotic disorders, bipolar disorders, or depressive disorders between 2003 and 2017 who had no diagnostic codes for these diseases, hypertension, diabetes, or dyslipidemia in 2002. The sample population comprised 712,950 subjects with major psychiatric disorders after random sampling 20% of the total, stratified by sex, age, region, and income classes.

From this sample population, people with depression were extracted as the target population for this study. A total of 569,321 subjects were diagnosed with depression and prescribed antidepressants, and 136,384 subjects were diagnosed with depression but not prescribed antidepressants between 1 January 2003, and 31 December 2017. The diagnosis of depression (F32 and F33) was defined according to the International Statistical Classification of Disease and Related Health Problems 10th revision (ICD-10). Diagnostic codes were entered by physicians when they saw patients. The use of antidepressants was defined as having a history of prescribing oral antidepressants from the initial diagnosis of depression to the follow-up period. We excluded those with incomplete baseline information, those who were diagnosed with hypertension, any diabetes, or dyslipidemia within 1 year before the diagnosis of depression, those under the age of 18, and those who were diagnosed with bipolar disorder with a primary diagnostic code. Finally, 276,048 subjects who were diagnosed with depression (DEP) and prescribed antidepressants (“DEP with antidepressants” group) and 79,119 subjects who were diagnosed with depression but not prescribed antidepressants (“DEP without antidepressants” group) were eligible to be included in the analyses. Their medical records until 31 December 2018 were reviewed.

We also set a healthy control (HC) group. A total of 405,111 subjects not diagnosed with any major psychiatric disorder during the study period and who were not diagnosed with hypertension, diabetes, or dyslipidemia in 2002 were included in the HC group. Among them, 276,048 subjects who met the eligibility criteria were matched with subjects in the group of DEP and antidepressants by exact age and sex (Supplementary Figure S1).

### Outcomes

The primary endpoint was newly diagnosed type 2 diabetes (ICD-10 codes of E11–E14 and prescription history of diabetes medications).

### Covariates

Income levels at baseline were divided into quartiles according to payment of health insurance. To consider the effect of individuals’ comorbidity, the Charlson comorbidity index (CCI) (27) was calculated by weighting pre-established diagnosis (dementia, pulmonary disease, connective tissue disorder, peptic ulcer, liver disease, paraplegia, renal disease, cancer, metastatic cancer, severe liver disease, and human immunodeficiency virus infection), excluding the diagnosis of metabolic and cardiovascular diseases corresponding to exclusion criteria of subject identification. Disabilities included mental disability, intellectual disability, and physical disability that were caused by a brain lesion, visual disability, and hearing impairment, and speech impairment. As psychiatric factors comorbid personality disorders, psychiatric medication use (antipsychotics, benzodiazepines, stimulants, mood stabilizers, and zolpidem), psychotic symptoms (F32.3 and F33.3), recurrent depression (F33), symptoms severity (mild: F32.0 and F33.0; moderate: F32.1 and F33.1; severe: F32.2, F32.3, F33.2, and F33.3), and history of admission to psychiatry (claim for at least one hospitalization) were identified. CCI, disability, and psychiatric factors were identified based on all claims during the observation period.

### Statistical analysis

Continuous variables are presented as mean ± standard deviation (SD), and categorical variables are presented as numbers and percentages. Analysis of variance (ANOVA) and chi-square tests were used to compare differences in factors between the groups. Cox proportional hazards regression analyses were conducted to identify the risk of type 2 diabetes by groups and censored by the occurrence of type 2 diabetes or death. The index date in depression patients with or without antidepressants was the date of the first occurrence of the diagnostic code for depressive disorder. In HCs, it was defined as the date of the first claim through a visit to a medical institution under any diagnostic code during the observation period. The proportional assumption was assessed using log–log survival plots. Hazard ratios (HRs) and corresponding 95% confidence intervals (CIs) are presented to show the magnitude of the risk of type 2 diabetes according to groups or variables. Analysis models were classified according to adjustment of variables, including crude (non-adjusted), Model 1 (adjusted for age and sex), Model 2 (adjusted for age, sex, and CCI), and Model 3 (adjusted for age, sex, CCI, income, disability, personality disorders, antipsychotic use, benzodiazepine use, stimulant use, mood stabilizer use, and zolpidem use). Sensitivity analyses including analyses within various groups, which were divided according to classes of antidepressants or detailed antidepressant ingredients, were performed. All statistical analyses were performed using SAS version 9.4 (SAS Institute Inc., Cary, NC, United States).

## Results

### Characteristics of study subjects

Table 1 shows the characteristics of the study subjects. The proportion of women in each group was distributed between 65% and 67%. The mean age at initial diagnosis of depression was 39.1 years (SD, 14.6 years) for the group of DEP without antidepressants, which was younger than that (39.9 years, SD, 13.9 years) of the group of DEP with antidepressants group and HCs (*p* < 0.001). Compared to HCs, the group of DEP with antidepressants and the group of DEP without antidepressants showed higher scores of CCI (*p* < 0.001). Compared to the group of DEPs without antidepressants, the group of DEP with antidepressants showed a higher proportion of all kinds of psychiatric medication use, psychotic symptoms, and admission to psychiatry. Regarding the symptom severity, the proportion of those with moderate or severe symptoms was higher in the group of DEP with antidepressants, while the proportion of those with mild symptoms was higher in the group of DEP without antidepressants.

**Table 1:** Baseline characteristics of the study subjects.

	DEP with antidepressants (*N*=276,048)	DEP without antidepressants (*N*=79,119)	Healthy controls (*N*=276,048)	Bonferroni corrected P		
				DEP with antidepressants vs. DEP without antidepressants	DEP with antidepressants vs. Healthy controls	DEP without antidepressants vs. Healthy controls
Sex				<0.001	1	<0.001
Male	91,997 (33.3)	27,735 (35.1)	91,997 (33.3)			
Female	184,051 (66.7)	51,384 (65.0)	184,051 (66.7)			
Age (years)	39.9 ± 13.9	39.1 ± 14.6	39.9 ± 13.9	<0.001	1	<0.001
Age group				<0.001	1	<0.001
18–39 years	144,487 (52.3)	44,279 (56.0)	144,487 (52.3)			
40–64 years	115,835 (42.0)	30,078 (38.0)	115,835 (42.0)			
≥65 years	15,726 (5.7)	4,762 (6.0)	15,726 (5.7)			
Income				0.007	<0.001	<0.001
Q1 (lowest)	70,480 (25.5)	19,828 (25.1)	68,050 (24.7)			
Q2	57,521 (20.8)	16,380 (20.7)	62,388 (22.6)			
Q3	66,842 (24.2)	19,208 (24.3)	72,144 (26.1)			
Q4 (highest)	81,205 (29.4)	23,703 (30.0)	73,466 (26.6)			
Charlson comorbidity index				<0.001	<0.001	<0.001
0	181,856 (65.9)	58,466 (73.9)	268,639 (97.3)			
1	57,281 (20.8)	14,936 (18.9)	6,379 (2.3)			
2	21,789 (7.9)	4,103 (5.2)	772 (0.3)			
≥3	15,122 (5.5)	1,614 (2.0)	258 (0.1)			
Disability	13,555 (4.9)	3,929 (5.0)	6,268 (2.3)	0.526	<0.001	<0.001
Personality disorders	9,229 (3.3)	1,447 (1.8)	342 (0.1)	<0.001	<0.001	<0.001
Antipsychotic use	27,154 (9.8)	6,356 (8.0)	63 (0.0)	<0.001	<0.001	<0.001
Benzodiazepine use	186,978 (67.7)	41,427 (52.4)	12,602 (4.6)	<0.001	<0.001	<0.001
Stimulant use	4,718 (1.7)	1,123 (1.4)	701 (0.3)	<0.001	<0.001	<0.001
Mood stabilizer use	12,011 (4.4)	2,340 (3.0)	321 (0.1)	<0.001	<0.001	<0.001
Zolpidem use	42,768 (15.5)	6,515 (8.2)	98 (0.0)	<0.001	<0.001	<0.001
Psychotic symptoms	3,448 (1.3)	947 (1.2)	0 (0.00)	0.251	<0.001	<0.001
Recurrent depression	18,324 (6.6)	5,131 (6.5)	0 (0.00)	0.129	<0.001	<0.001
Symptom severity				<0.001	<0.001	<0.001
Mild	64,648 (23.4)	21,445 (27.1)	0 (0.0)			
Moderate	59,984 (21.7)	12,941 (16.4)	0 (0.0)			
Severe	19,906 (7.2)	4,391 (5.6)	0 (0.0)			
Other	131,510 (47.6)	40,342 (51.0)	0 (0.0)			
Admission to psychiatry	57,94 (2.1)	1,165 (1.5)	110 (0.0)	<0.001	<0.001	<0.001

DEP, depression. Data are expressed as the mean ± standard deviation, or *n* (%).

### Incidence of type 2 diabetes

In the group of DEP with antidepressants, during an average follow-up period of 8.5 years (SD, 4.7 years), 14,860 subjects were newly diagnosed with type 2 diabetes with an incidence rate of 6.5 per 1,000 person-years. Among those in the group of DEP without antidepressants, during an average follow-up period of 7.8 years (SD, 4.8 years), 2,898 subjects were newly diagnosed with type 2 diabetes with an incidence rate of 4.8 per 1,000 person-years. Among HCs, during an average follow-up period of 14.9 years (SD, 3.2 years), 24,577 subjects were newly diagnosed with type 2 diabetes with an incidence rate was 6.2 per 1,000 person-years. The incidence rate tended to increase with age (Supplementary Figure S2).

### Risk of type 2 diabetes by groups

Table 2 presents HRs and 95% CIs for type 2 diabetes according to groups. In the crude model, compared to HCs, the group of DEP with antidepressants showed an increased risk of type 2 diabetes (HR, 1.16; 95% CI, 1.14–1.19), and the group of DEP without antidepressants showed a decreased risk (HR, 0.86; 95% CI, 0.83–0.90). However, in the fully adjusted model, compared to HCs, both the group of DEP with antidepressants (adjusted hazard ratio [aHR], 0.86; 95% CI, 0.83–0.88) and the group of DEP without antidepressants (aHR 0.78; 95% 0.74–0.81) showed a decreased risk of type 2 diabetes.

**Table 2:** Hazard ratios and 95% confidence interval on type 2 diabetes according to groups.

	Subjects (*N*)	Events (*N*)	Follow-up duration (person-year)	Incidence rate (per 1000 person-years)	Hazard ratio (95% Confidence interval)			
					Crude	Model 1^a^	Model 2^b^	Model 3^c^
Healthy controls	276,048	24,577	3,954,318	6.2	1 (Ref.)	1 (Ref.)	1 (Ref.)	1 (Ref.)
DEP with antidepressants	276,048	14,860	2,283,663	6.5	1.16 (1.14–1.19)^***^	1.06 (1.04–1.08)^***^	0.95 (0.93–0.98)^***^	0.86 (0.83–0.88)^***^
DEP without antidepressants	79,119	2,898	603,168	4.8	0.86 (0.83–0.90)^***^	0.88 (0.85–0.92)^***^	0.85 (0.82–0.88)^***^	0.78 (0.74–0.81)^***^

DEP, depression. ^*^p <0.05, ^**^p <0.01, ^***^p <0.001.

a Adjusted for age (years) and sex.

b Adjusted for age (years), sex, and Charlson comorbidity index.

c Adjusted for age (years), sex, Charlson comorbidity index, income, disability, personality disorders, antipsychotic use, benzodiazepine use, stimulant use, mood stabilizer use, and zolpidem use.

Table 3 presents HRs and 95% CIs according to age groups categorized as 18–39 years, 40–64 years, and ≥ 65 years. Among those aged 18–39 years, compared to HCs, the group of DEP with antidepressants (aHR, 1.24; 95% CI, 1.16–1.32) and the group of DEP without antidepressants (aHR, 1.17; 95% CI 1.08–1.27) showed increased risks for type 2 diabetes after full adjustment. Among those aged 40–64 years, compared to HCs, both the group of DEP with antidepressants (aHR, 0.81; 95% CI, 0.78–0.84) and the group of DEP without antidepressants (aHR, 0.74; 95% CI, 0.70–0.78) showed decreased risks of type 2 diabetes after full adjustment. Among those aged ≥65 years, compared to HCs, both the group of DEP with antidepressants (aHR, 0.69; 95% CI, 0.63–0.75) and the group of DEP without antidepressants (aHR, 0.70; 95% CI, 0.62–0.78) showed decreased risks of type 2 diabetes after full adjustment.

**Table 3:** Hazard ratios and 95% confidence interval on type 2 diabetes according to the age group.

Age group	Group	Subjects (*N*)	Events (*N*)	Follow-up duration (person-year)	Incidence rate (per 1000 person-years)	Hazard ratio (95% Confidence interval)			
						Crude	Model 1^a^	Model 2^b^	Model 3^c^
18–39 years	Healthy controls	144,487	5,302	2,170,803	2.4	1 (Ref.)	1 (Ref.)	1 (Ref.)	1 (Ref.)
	DEP with antidepressants	144,487	3,197	1,135,441	2.8	1.59 (1.52–1.67)^***^	1.54 (1.47–1.61)^***^	1.40 (1.34–1.48)^***^	1.24 (1.16–1.32)^***^
	DEP without antidepressants	44,279	837	349,550	2.4	1.34 (1.24–1.44)^***^	1.33 (1.23–1.43)^***^	1.28 (1.19–1.38)^***^	1.17 (1.08–1.27)^***^
40–64 years	Healthy controls	115,835	16,286	1,622,580	10.0	1 (Ref.)	1 (Ref.)	1 (Ref.)	1 (Ref.)
	DEP with antidepressants	115,835	9,587	1,012,624	9.5	1.02 (1.00–1.05)	1.02 (0.99–1.04)	0.89 (0.86–0.92)^***^	0.81 (0.78–0.84)^***^
	DEP without antidepressants	30,078	1,674	227,041	7.4	0.80 (0.77–0.85)^***^	0.83 (0.79–0.88)^***^	0.80 (0.76–0.84)^***^	0.74 (0.70–0.78)^***^
≥65 years	Healthy controls	15,726	2,989	160,935	18.6	1 (Ref.)	1 (Ref.)	1 (Ref.)	1 (Ref.)
	DEP with antidepressants	15,726	2,076	135,598	15.3	0.80 (0.76–0.85)^***^	0.80 (0.76–0.85)^***^	0.71 (0.67–0.77)^***^	0.69 (0.63–0.75)^***^
	DEP without antidepressants	4,762	387	26,577	14.6	0.75 (0.67–0.83)^***^	0.75 (0.67–0.84)^***^	0.73 (0.65–0.81)^***^	0.70 (0.62–0.78)^***^

DEP, depression. ^*^p <0.05, ^**^p <0.01, ^***^p <0.001.

a Adjusted for age (years) and sex.

b Adjusted for age (years), sex, and Charlson comorbidity index.

c Adjusted for age (years), sex, Charlson comorbidity index, income, disability, personality disorders, antipsychotic use, benzodiazepine use, stimulant use, mood stabilizer use, and zolpidem use.

### Risk of type 2 diabetes by antidepressant use

Table 4 shows HRs and 95% CIs according to combinations of antidepressant use and DEP without antidepressants on type 2 diabetes compared to HCs. In the fully adjusted model, compared to HCs, those who had used tricyclic antidepressants (TCAs) only (aHR, 1.25; 95% CI, 1.20–1.30) showed increased risks of type 2 diabetes. The remaining groups showed decreased risks of type 2 diabetes.

**Table 4:** Hazard ratios and 95% confidence intervals of antidepressant combinations and DEP without antidepressants group on type 2 diabetes compared to healthy controls.

	Subjects (*n*)	Events (*n*)	Follow-up duration (person-year)	Incidence rate (per 1000 person-years)	Hazard ratio (95% confidence interval)			
					Crude	Model 1^a^	Model 2^b^	Model 3^c^
Healthy controls	276,048	24,577	3,954,318	6.2	1 (Ref.)	1 (Ref.)	1 (Ref.)	1 (Ref.)
DEP without antidepressants	79,119	2,898	603,168	4.8	0.86 (0.83–0.90)^***^	0.88 (0.85–0.92)^***^	0.85 (0.82–0.88)^***^	0.78 (0.74–0.81)^***^
SSRI only	70,108	2,117	455,491	4.6	0.85 (0.81–0.89)^***^	1.07 (1.02–1.12)^**^	1.02 (0.97–1.07)	0.86 (0.81–0.90)^***^
TCA only	48,754	5,098	439,207	11.6	2.04 (1.98–2.11)^***^	1.61 (1.56–1.66)^***^	1.41 (1.37–1.46)^***^	1.25 (1.20–1.30)^***^
SSRI+TCA	41,358	2,275	413,002	5.5	0.97 (0.93–1.01)	0.89 (0.85–0.93)^***^	0.77 (0.74–0.81)^***^	0.66 (0.63–0.70)^***^
Other combinations	115,828	5,370	975,963	5.5	0.97 (0.95–1.00)	0.85 (0.82–0.87)^***^	0.73 (0.71–0.75)^***^	0.60 (0.58–0.63)^***^

DEP, depression; SSRI, selective serotonin reuptake inhibitors; TCA, tricyclic antidepressant. ^*^p <0.05, ^**^p <0.01, ^***^p <0.001.

a Adjusted for age (years) and sex.

b Adjusted for age (years), sex, and Charlson comorbidity index.

c Adjusted for age (years), sex, Charlson comorbidity index, income, disability, personality disorders, antipsychotic use, benzodiazepine use, stimulant use, mood stabilizer use, and zolpidem use.

Supplementary Tables S1–S6 show results of sensitivity analyses including analyses within various groups divided according to classes of antidepressants or detailed antidepressant components. Compared to HCs, the risk of type 2 diabetes was generally reduced in patients with depression regardless of the use of antidepressants or the type of drugs. However, the use of TCAs was associated with an increased risk of type 2 diabetes compared to the DEP without antidepressants group.

### Subgroup analysis

We performed a subgroup analysis for depression patients with diagnostic codes having symptom severity information. A total of 144,538 (52.4%) participants in the group of DEP with antidepressants and 38,608 (49.0%) participants in the group of DEP without antidepressants had diagnostic codes with symptom severity information. In the regression model additionally adjusted for symptom severity, risks of type 2 diabetes for the group of DEP with antidepressants (aHR, 0.81; 95% CI: 0.77–0.85) and the group of DEP without antidepressants (aHR, 0.74; 95% CI: 0.69–0.79) compared to HCs were similar to the main outcomes (Supplementary Table S7).

## Discussion

In this study, we examined associations of depression and antidepressant use with the occurrence of newly diagnosed type 2 diabetes. As a result, after adjustments, the overall risk of type 2 diabetes was lowered in patients with depression compared to that in HCs regardless of the use of antidepressants. However, the risk considerably varied according to the age of patients, comorbid physical diseases, and type of antidepressants used.

This study found that the risk of type 2 diabetes was lower in patients with depression than in HCs, which is inconsistent with previous studies. The difference seemed to be largely attributable to comorbid metabolic disorders. In this study, people with a previous history of hypertension or dyslipidemia besides diabetes were excluded from subject identification. Hypertension and dyslipidemia together with abdominal obesity are components that constitute the metabolic syndrome, sharing underlying mechanisms such as insulin resistance and genetic predisposition. It is widely accepted that they can increase the risk of diabetes (28, 29). Considering their high prevalence and the fact that depression often accompanies people with these diseases (30), hypertension and dyslipidemia can act as strong confounders in the relationship between depression and type 2 diabetes. Therefore, we tried to identify the association between depression and type 2 diabetes in relatively metabolically healthy subjects by excluding those who had a diagnostic history of these diseases before the index date. This methodology seemed to have caused different results compared to previous studies.

Moreover, among depression patients with antidepressants, Cox regression analyses showed increased risks of type 2 diabetes in the crude model and the model adjusted for age and sex. However, the risk was decreased when physical comorbidity was additionally adjusted. This suggests that comorbid illness can increase the risk of type 2 diabetes, rather than antidepressants themselves being a direct risk factor for type 2 diabetes in patients with depression taking antidepressants. Although depression and antidepressants do not substantially increase the risk of type 2 diabetes, the high comorbidity of depression in other physical illnesses (which are risk factors for type 2 diabetes) might have made it appear as if depression and antidepressants were risk factors. Moreover, in the fully adjusted model, when other factors such as psychiatric comorbidity (i.e., personality disorder) and other psychiatric medication use were additionally included, the risk of type 2 diabetes was further decreased both in the group of DEP with antidepressants and the group of DEP without antidepressants. This suggests that other psychiatric conditions and medications could also affect the risk of type 2 diabetes.

As an explanation for the decreased risk of type 2 diabetes among depression patients, changes in appetite or weight, which are symptoms of depression, could affect the results of this study. While the manifestation of symptom clusters is heterogeneous among patients with depression, they often experience decreased appetite and weight loss, which could be protective against type 2 diabetes. However, the risk of type 2 diabetes varies depending on the age at the onset of depression. In young adults, the risk increased even after adjusting for CCI and other potential risk factors for type 2 diabetes, which was contrary to the result for those aged ≥40 years. To explain this, two aspects can be considered. First, young adults with depression correspond to having early-onset depression. Earlier onset age is known to be associated with a higher genetic burden (31). A previous study has reported that young people at increased familial risk of depression are likely to have diminished insulin sensitivity even without depressive symptoms, suggesting vulnerability to diabetes (32). In addition, young adults are more likely to have depression with atypical features, which shows the reverse of neurovegetative symptoms such as increased appetite and weight gain while those with other types commonly have a poor appetite and weight loss as their depressive symptoms (33). These metabolically opposite manifestations of depressive symptoms between young adult patients and older patients might have led to opposite outcomes.

In all age groups, the increased risk due to taking antidepressants was not significantly different from the risk in the group not taking antidepressants. In fully adjusted models, the difference ranged only 1%–8%. However, among those with depression, compared to those without antidepressants, the risk varied depending on the class of antidepressants. In particular, the risk of type 2 diabetes was reduced in those who had used only SSRIs, but the risk was increased in those who had used only TCAs. The weight gain liability of each antidepressant or clinical aspects considered when they were prescribed might have affected these results. However, since this study did not include the dose or duration of the drug used, further research is needed to examine the causal inference.

Caution is needed when interpreting the results of the present study showing that depression is a preventive factor for type 2 diabetes, especially in those after middle age. Although a decreased risk was observed when potential confounders that could affect the relationship between depression and type 2 diabetes were corrected, in the crude model, especially in people who had used antidepressants, an increased risk was observed compared to that for HCs. Moreover, the subsequent risk for type 2 diabetes could be higher if we also considered those who previously had metabolic diseases such as hypertension or dyslipidemia. Therefore, while we found that having depression itself does not lead to a high risk for type 2 diabetes, it should be noted that the occurrence of subsequent diabetes can often be observed in clinical settings considering the high comorbidity of depression in other physical illnesses which are associated with type 2 diabetes. Rather, if a patient with depression has many metabolic risk factors such as obesity, hypertension, and hyperlipidemia, more clinical attention should be paid to the detection of diabetes since depressive symptoms such as decreased motivation or energy level can prevent them from participating in health-promoting behaviors such as health checkups or seeking adequate physical care even if they have symptoms or signs of diabetes.

This study provides novel findings that depression and antidepressants are generally associated with a decreased risk of type 2 diabetes after adjusting for other physical comorbidities rather than being a risk factor as described in the results of previous studies. On the other hand, the increase in risk was noteworthy in young adult patients with depression. Compared to the group not taking antidepressants, there was a difference in the risk according to classes of antidepressants. These results suggest that an individualized approach that considers age at onset, physical comorbidity, and other metabolic risk factors is needed for screening and detecting type 2 diabetes rather than viewing those with depression as a population at high risk.

This study has some limitations. First, the diagnosis of depression was identified only through diagnostic codes without any structured evaluation because we used claims data. However, in South Korea, a patient can see a specialist without a referral from a primary care physician. Therefore, it is assumed that most of the initial diagnosis of depression was made by psychiatrists. Second, since the depression group included only cases diagnosed by visiting a medical institute, there might be people who met the criteria for the depressive disorder but did not visit a hospital who might have been included in HCs. Third, because the data source included claims data from 2002 with a clean period set as 1 year, those who had previously been diagnosed with depression but did not visit a medical institution in 2002 (the clean period) and then visited again for depression that could be considered as new-onset depression were included in the analyses. Moreover, the possibility of such bias would increase as the age of subjects increased. This should be considered particularly when interpreting results according to age group. Fourth, we extracted and matched HCs based only on DEP with the antidepressant group. There was no separate matched control group based on the group of DEP without antidepressants. Due to the lack of matching in the DEP without antidepressants group, age and sex were included as adjusting variables in the analysis models. Moreover, matching was done based on data in 2002. Thus, time-related bias might have occurred due to differences in index year and follow-up period between patients with depression and HCs. Fifth, while we tried to include factors that could affect the relationship between depression, antidepressants, and type 2 diabetes in our analyses, some factors such as family history, lifestyle factors, overweight/obesity, and polycystic ovarian syndrome were not included in the analyses. Moreover, regarding the use of antidepressants, we focused on whether antidepressants were used and the classes or ingredients of antidepressants. However, other clinical factors related to antidepressant use (e.g., the timing of the antidepressant exposure, the use of multiple antidepressants, changes in dose, or drug discontinuation) could also affect the relationship between depression and the occurrence of type 2 diabetes. They should be the focus of future research. In addition, while the severity of depressive symptoms could affect the use of antidepressants, it was included only in the subgroup analyses, but not in the main analyses since only about half of the subjects in depression groups had severity information in their diagnostic codes. Finally, we excluded those who had hypertension or dyslipidemia at baseline and 1 year before the index date considering their potentially strong confounding effects because these diseases are strong risk factors for type 2 diabetes and the prevalence of depression is high in people with these diseases. Although excluding those with these conditions was to examine more direct effects of depression and antidepressants on type 2 diabetes in a relatively metabolically healthy population, caution is needed when interpreting our results and applying them to real-world clinical settings.

In conclusion, in this nationally representative cohort study, we found that depression and antidepressant medications *per se* were not contributory factors for type 2 diabetes. Clinical attention is needed for patients with depression for the detection of type 2 diabetes, especially in those with early age at onset, those with physical comorbidity, and those with metabolic risk factors.

## Data availability statement

Publicly available datasets were analyzed in this study. This data can be found here: https://nhiss.nhis.or.kr/.

## Ethics statement

The studies involving humans were approved by Institutional Review Board of Samsung Medical Center. The studies were conducted in accordance with the local legislation and institutional requirements. The ethics committee/institutional review board waived the requirement of written informed consent for participation from the participants or the participants’ legal guardians/next of kin because As it involved retrospective analyses of de-identified data.

## Author contributions

HK: Conceptualization, Methodology, Writing – original draft, Writing – review & editing. Y-BL: Methodology, Writing – review & editing. JL: Formal analysis, Methodology, Visualization, Writing – review & editing. DK: Writing – review & editing. GK: Writing – review & editing. S-MJ: Writing – review & editing. JK: Writing – review & editing. KH: Conceptualization, Methodology, Project administration, Writing – review & editing. HJ: Conceptualization, Methodology, Project administration, Writing – review & editing.
