# Supplementary material for: Depression, antidepressant use, and the risk of type 2 diabetes: a nationally representative cohort study
Source: Front Psychiatry. 2023 Dec 6;14:1275984. doi: 10.3389/fpsyt.2023.1275984 (PMC10731300; doi:10.3389/fpsyt.2023.1275984)
Supplement: Supplementary file 1 [file Data_Sheet_1.docx]

**Supplementary Figure 1. Flow chart showing the identification of subjects.**


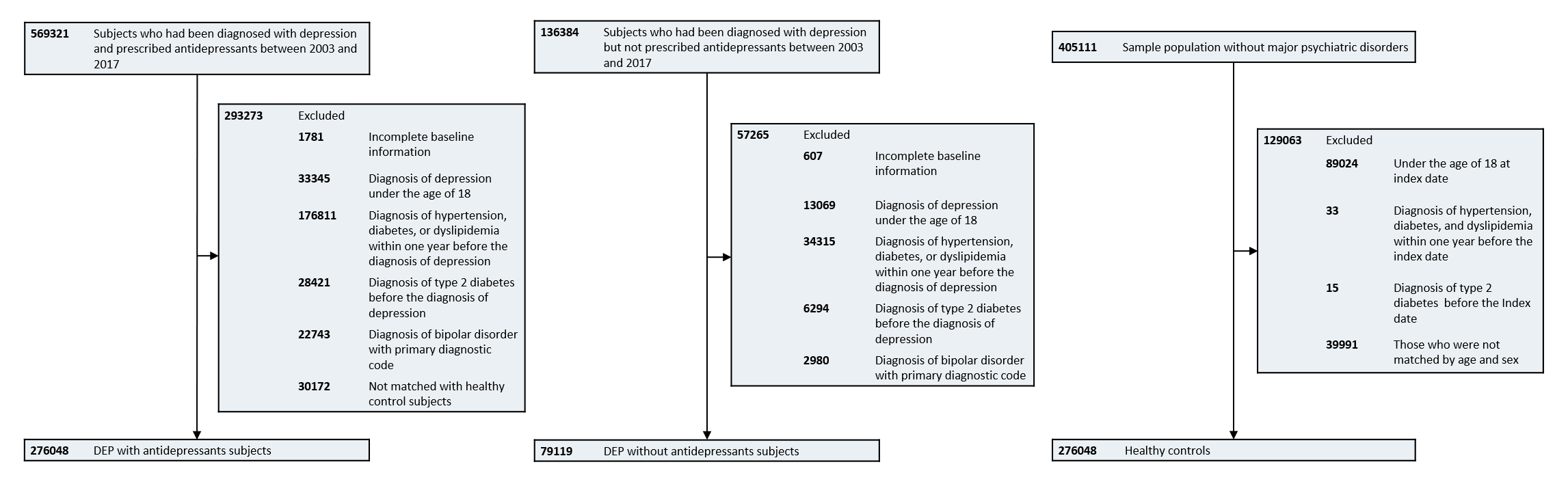


**Supplementary Figure 2. Kaplan–Meier curves for type 2 diabetes according to the group**

(a) All subjects; (b) 18–39 years; (c) 40–64 years; (d) ≥65 years


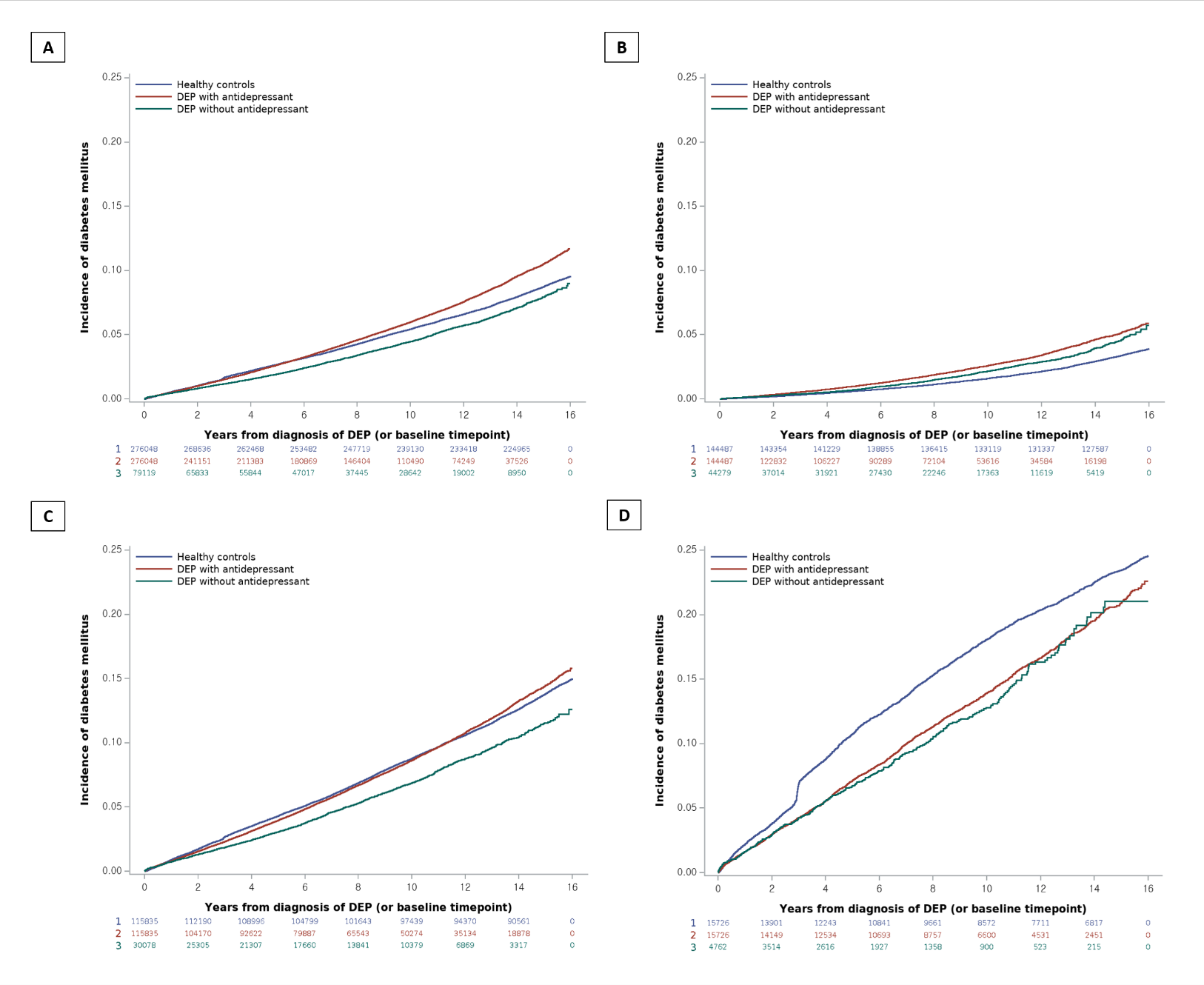


**Supplementary Table 1. Hazard ratios and 95% confidence intervals** **of antidepressant classes on type 2 diabetes compared to healthy controls**

|  | **Subjects**  **(n)** | **Events (n)** | **Follow-up duration (person-year)** | **Incidence rate**  **(per 1000 person-years)** | **Hazard ratio (95% Confidence interval)** | | | |
| --- | --- | --- | --- | --- | --- | --- | --- | --- |
|  |  |  |  |  | **Crude** | **Model 1^a^** | **Model 2^b^** | **Model 3^c^** |
| Healthy controls | 276,048 | 24,577 | 3,954,318 | 6.2 | 1 (Ref.) | 1 (Ref.) | 1 (Ref.) | 1 (Ref.) |
| SSRI | 188,456 | 7,557 | 1,542,828 | 4.9 | 0.88 (0.86–0.91)^***^ | 0.87 (0.85–0.90)^***^ | 0.88 (0.85–0.90)^***^ | 0.76 (0.73–0.79)^***^ |
| SNRI | 46,991 | 2,039 | 422,692 | 4.8 | 0.85 (0.81–0.89)^***^ | 0.72 (0.69–0.75)^***^ | 0.69 (0.66–0.73)^***^ | 0.60 (0.56–0.64)^***^ |
| TCA | 151,588 | 10,907 | 1,483,952 | 7.3 | 1.29 (1.26–1.32)^***^ | 1.06 (1.03–1.08)^***^ | 0.91 (0.88–0.93)^***^ | 0.82 (0.79–0.85)^***^ |
| MAOI | 316 | 30 | 3,355 | 8.9 | 1.54 (1.07–2.19)^*^ | 0.62 (0.43–0.89)^**^ | 0.50 (0.35–0.73)^**^ | 0.45 (0.31–0.65)^***^ |
| Others | 90,009 | 4,170 | 749,392 | 5.6 | 0.99 (0.95–1.02) | 0.86 (0.83–0.89)^***^ | 0.83 (0.80–0.86)^***^ | 0.67 (0.64–0.71)^***^ |

SSRI, selective serotonin reuptake inhibitors; SNRI, serotonin-norepinephrine reuptake inhibitor; TCA, tricyclic antidepressant; MAOI, monoamine oxidase inhibitor

**^a^**Adjusted for age (years) and sex

**^b^**Adjusted for age (years), sex, and Charlson comorbidity Index

**^c^**Adjusted for age (years), sex, Charlson comorbidity index, income, disability, personality disorders, antipsychotic use, benzodiazepine use, stimulant use, mood stabilizer use, and zolpidem use

^*^*P* <0.05, ^**^*P* <0.01, ^***^*P* <0.001

**Supplementary Table 2. Hazard ratios and 95% confidence intervals of antidepressant classes on type 2 diabetes compared to DEP without antidepressants group.**

|  | **Subjects**  **(n)** | **Events (n)** | **Follow-up duration (person-year)** | **Incidence rate**  **(per 1000 person-years)** | **Hazard ratio (95% Confidence interval)** | | | |
| --- | --- | --- | --- | --- | --- | --- | --- | --- |
|  |  |  |  |  | **Crude** | **Model 1^a^** | **Model 2^b^** | **Model 3^c^** |
| DEP without antidepressants | 79,119 | 2,898 | 603,168 | 4.8 | 1 (Ref.) | 1 (Ref.) | 1 (Ref.) | 1 (Ref.) |
| SSRI | 188,456 | 7,557 | 1,542,828 | 4.9 | 1.02 (0.97–1.06) | 0.98 (0.93–1.02) | 0.97 (0.93–1.02) | 0.94 (0.90–0.98)^**^ |
| SNRI | 46,991 | 2,039 | 422,692 | 4.8 | 0.97 (0.92–1.03) | 0.82 (0.78–0.87)^***^ | 0.81 (0.76–0.86)^***^ | 0.78 (0.74–0.83)^***^ |
| TCA | 151,588 | 10,907 | 1,483,952 | 7.3 | 1.48 (1.42–1.55)^***^ | 1.23 (1.18–1.28)^***^ | 1.11 (1.06–1.16)^***^ | 1.09 (1.05–1.14)^***^ |
| MAOI | 316 | 30 | 3,355 | 8.9 | 1.76 (1.23–2.53)^**^ | 0.79 (0.55–1.13) | 0.78 (0.54–1.12) | 0.80 (0.56–1.16) |
| Others | 90,009 | 4,170 | 749,392 | 5.6 | 1.14 (1.08–1.19)^***^ | 0.99 (0.94–1.03) | 0.96 (0.92–1.01) | 0.90 (0.85–0.94)^***^ |

DEP, depression; SSRI, selective serotonin reuptake inhibitors; SNRI, serotonin-norepinephrine reuptake inhibitor; TCA, tricyclic antidepressant; MAOI, monoamine oxidase inhibitor

**^a^**Adjusted for age (years) and sex

**^b^**Adjusted for age (years), sex, and Charlson comorbidity Index

**^c^**Adjusted for age (years), sex, Charlson comorbidity index, income, disability, personality disorders, antipsychotic use, benzodiazepine use, stimulant use, mood stabilizer use, and zolpidem use

^*^*P* <0.05, ^**^*P* <0.01, ^***^*P* <0.001

**Supplementary Table 3. Hazard ratios and 95% confidence intervals of use of antidepressants on type 2 diabetes compared to healthy controls**

|  |  | **Subjects**  **(n)** | **Events (n)** | **Follow-up duration (person-year)** | **Incidence rate**  **(per 1000 person-years)** | **Hazard ratio (95% Confidence interval)** | | | |
| --- | --- | --- | --- | --- | --- | --- | --- | --- | --- |
|  |  |  |  |  |  | **Crude** | **Model 1^a^** | **Model 2^b^** | **Model 3^c^** |
| Healthy controls | | 276,048 | 24,577 | 3,954,318 | 6.2 | 1 (Ref.) | 1 (Ref.) | 1 (Ref.) | 1 (Ref.) |
| SSRI | |  |  |  |  |  |  |  |  |
|  | Escitalopram | 113,437 | 3,762 | 870,521 | 4.3 | 0.78 (0.75–0.81)^***^ | 0.75 (0.72–0.77)^***^ | 0.73 (0.70–0.76)^***^ | 0.63 (0.59–0.66)^***^ |
|  | Fluoxetine | 36,605 | 1,265 | 318,606 | 4.0 | 0.70 (0.66–0.74)^***^ | 0.91 (0.86–0.97)^**^ | 0.88 (0.83–0.93)^***^ | 0.78 (0.73–0.84)^***^ |
|  | Fluvoxamine | 10,165 | 540 | 109,441 | 4.9 | 0.85 (0.78–0.92)^**^ | 0.85 (0.78–0.93)^***^ | 0.77 (0.70–0.85)^***^ | 0.68 (0.61–0.76)^***^ |
|  | Sertraline | 47,174 | 2,149 | 434,648 | 4.9 | 0.86 (0.83–0.90)^***^ | 0.85 (0.81–0.88)^***^ | 0.84 (0.80–0.88)^***^ | 0.74 (0.69–0.79)^***^ |
|  | Paroxetine | 50,753 | 2,495 | 491,563 | 5.1 | 0.89 (0.85–0.92)^***^ | 0.89 (0.85–0.92)^***^ | 0.86 (0.82–0.90)^***^ | 0.76 (0.71–0.81)^***^ |
| SNRI | |  |  |  |  |  |  |  |  |
|  | Duloxetine | 21,341 | 836 | 190,708 | 4.4 | 0.77 (0.72–0.82)^***^ | 0.56 (0.53–0.60)^***^ | 0.52 (0.48–0.56)^***^ | 0.45 (0.41–0.50)^***^ |
|  | Venlafaxine | 19,774 | 852 | 181,724 | 4.7 | 0.82 (0.76–0.87)^***^ | 0.83 (0.78–0.89)^***^ | 0.78 (0.73–0.84)^***^ | 0.68 (0.62–0.74)^***^ |
|  | Desvenlafaxine | 2,847 | 30 | 16,584 | 1.8 | 0.32 (0.22–0.45)^***^ | 0.35 (0.24–0.49)^***^ | 0.28 (0.20–0.41)^***^ | 0.24 (0.16–0.35)^***^ |
|  | Milnacipran | 9,514 | 578 | 99,676 | 5.8 | 1.00 (0.92–1.09) | 0.82 (0.75–0.89)^***^ | 0.73 (0.66–0.80)^***^ | 0.64 (0.57–0.71)^***^ |
| TCA | |  |  |  |  |  |  |  |  |
|  | Nortriptyline | 49,428 | 3,038 | 495,514 | 6.1 | 1.07 (1.03–1.11)^**^ | 0.85 (0.81–0.88)^***^ | 0.75 (0.71–0.78)^***^ | 0.68 (0.64–0.72)^***^ |
|  | Clomipramine | 5,543 | 351 | 57,682 | 6.1 | 1.05 (0.94–1.16) | 1.09 (0.98–1.21) | 0.99 (0.88–1.10) | 0.86 (0.76–0.98)^*^ |
|  | Amitriptyline | 109,334 | 8,482 | 1,108,600 | 7.7 | 1.33 (1.30–1.37)^***^ | 1.03 (1.01–1.06)^*^ | 0.88 (0.85–0.91)^***^ | 0.79 (0.76–0.83)^***^ |
|  | Imipramine | 36,367 | 2,489 | 377,730 | 6.6 | 1.14 (1.09–1.19)^***^ | 1.00 (0.95–1.04) | 0.91 (0.87–0.96)^***^ | 0.82 (0.77–0.87)^***^ |
| MAOI | |  |  |  |  |  |  |  |  |
|  | Selegiline | 316 | 30 | 3,355 | 8.9 | 1.54 (1.07–2.19)^*^ | 0.62 (0.43–0.89)^**^ | 0.50 (0.35–0.73)^***^ | 0.45 (0.31–0.65)^***^ |
| Others | |  |  |  |  |  |  |  |  |
|  | Mirtazapine | 18,590 | 1,018 | 173,939 | 5.9 | 1.02 (0.95–1.08) | 0.80 (0.75–0.85)^***^ | 0.77 (0.72–0.83)^***^ | 0.63 (0.58–0.69)^***^ |
|  | Bupropion | 14,203 | 458 | 110,474 | 4.1 | 0.73 (0.67–0.80)^***^ | 0.77 (0.70–0.85)^***^ | 0.73 (0.66–0.80)^***^ | 0.62 (0.55–0.70)^***^ |
|  | Trazodone | 67,548 | 3,282 | 580,751 | 5.7 | 1.00 (0.96–1.03) | 0.87 (0.84–0.90)^***^ | 0.81 (0.78–0.85)^***^ | 0.67 (0.63–0.71)^***^ |
|  | Vortioxetine | 7,697 | 57 | 41,852 | 1.4 | 0.24 (0.18–0.31)^***^ | 0.24 (0.18–0.31)^***^ | 0.20 (0.16–0.27)^***^ | 0.17 (0.13–0.22)^***^ |

SSRI, selective serotonin reuptake inhibitors; SNRI, serotonin-norepinephrine reuptake inhibitor; TCA, tricyclic antidepressant; MAOI, monoamine oxidase inhibitor

**^a^**Adjusted for age (years) and sex

**^b^**Adjusted for age (years), sex, and Charlson comorbidity Index

**^c^**Adjusted for age (years), sex, Charlson comorbidity index, income, disability, personality disorders, antipsychotic use, benzodiazepine use, stimulant use, mood stabilizer use, and zolpidem use

^*^*P* <0.05, ^**^*P* <0.01, ^***^*P* <0.001

**Supplementary Table 4. Hazard ratios and 95% confidence intervals of use of antidepressants on type 2 diabetes compared to DEP without antidepressants group.**

|  |  | **Subjects**  **(n)** | **Events (n)** | **Follow-up duration (person-year)** | **Incidence rate**  **(per 1000 person-years)** | **Hazard ratio (95% Confidence interval)** | | | |
| --- | --- | --- | --- | --- | --- | --- | --- | --- | --- |
|  |  |  |  |  |  | **Crude** | **Model 1^a^** | **Model 2^b^** | **Model 3^c^** |
| DEP without antidepressants | | 79,119 | 2,898 | 603,168 | 4.8 | 1 (Ref.) | 1 (Ref.) | 1 (Ref.) | 1 (Ref.) |
| SSRI | |  |  |  |  |  |  |  |  |
|  | Escitalopram | 113,437 | 3,762 | 870,521 | 4.3 | 0.92 (0.88–0.97)^***^ | 0.85 (0.81–0.90)^***^ | 0.84 (0.80–0.89)^***^ | 0.81 (0.77–0.85)^***^ |
|  | Fluoxetine | 36,605 | 1,265 | 318,606 | 4.0 | 0.81 (0.76–0.87)^***^ | 0.98 (0.92–1.05) | 0.98 (0.91–1.05) | 0.96 (0.90–1.03) |
|  | Fluvoxamine | 10,165 | 540 | 109,441 | 4.9 | 0.98 (0.89–1.07) | 0.94 (0.86–1.04) | 0.92 (0.84–1.02) | 0.92 (0.83–1.01) |
|  | Sertraline | 47,174 | 2,149 | 434,648 | 4.9 | 1.00 (0.94–1.05) | 0.94 (0.89–1.00)^*^ | 0.95 (0.89–1.00) | 0.92 (0.87–0.97)^**^ |
|  | Paroxetine | 50,753 | 2,495 | 491,563 | 5.1 | 1.02 (0.96–1.07) | 0.98 (0.92–1.03) | 0.97 (0.92–1.02) | 0.94 (0.89–1.00)^*^ |
| SNRI | |  |  |  |  |  |  |  |  |
|  | Duloxetine | 21,341 | 836 | 190,708 | 4.4 | 0.89 (0.82–0.96)^**^ | 0.67 (0.62–0.72)^***^ | 0.65 (0.60–0.71)^***^ | 0.64 (0.59–0.70)^***^ |
|  | Venlafaxine | 19,774 | 852 | 181,724 | 4.7 | 0.95 (0.88–1.02) | 0.92 (0.85–0.99)^*^ | 0.91 (0.84–0.99)^*^ | 0.89 (0.82–0.96)^**^ |
|  | Desvenlafaxine | 2,847 | 30 | 16,584 | 1.8 | 0.38 (0.26–0.54)^***^ | 0.39 (0.27–0.56)^***^ | 0.38 (0.27–0.55)^***^ | 0.38 (0.26–0.55)^***^ |
|  | Milnacipran | 9,514 | 578 | 99,676 | 5.8 | 1.15 (1.05–1.26)^**^ | 0.94 (0.86–1.03) | 0.92 (0.83–1.00) | 0.91 (0.83–1.00)^*^ |
| TCA | |  |  |  |  |  |  |  |  |
|  | Nortriptyline | 49,428 | 3,038 | 495,514 | 6.1 | 1.22 (1.16–1.28)^***^ | 0.98 (0.93–1.03) | 0.91 (0.86–0.96)^***^ | 0.90 (0.85–0.95)^***^ |
|  | Clomipramine | 5,543 | 351 | 57,682 | 6.1 | 1.21 (1.09–1.36)^***^ | 1.19 (1.07–1.33)^**^ | 1.17 (1.05–1.31)^**^ | 1.13 (1.01–1.26)^*^ |
|  | Amitriptyline | 109,334 | 8,482 | 1,108,600 | 7.7 | 1.53 (1.47–1.60)^***^ | 1.21 (1.16–1.26)^***^ | 1.09 (1.04–1.14)^***^ | 1.08 (1.03–1.12)^**^ |
|  | Imipramine | 36,367 | 2,489 | 377,730 | 6.6 | 1.30 (1.23–1.37)^***^ | 1.13 (1.07–1.19)^***^ | 1.08 (1.02–1.14)^**^ | 1.06 (1.00–1.12) |
| MAOI | |  |  |  |  |  |  |  |  |
|  | Selegiline | 316 | 30 | 3,355 | 8.9 | 1.76 (1.23–2.53)^**^ | 0.79 (0.55–1.13) | 0.78 (0.54–1.12) | 0.80 (0.56–1.16) |
| Others | |  |  |  |  |  |  |  |  |
|  | Mirtazapine | 18,590 | 1,018 | 173,939 | 5.9 | 1.18 (1.10–1.26)^***^ | 0.93 (0.86–1.00)^*^ | 0.93 (0.86–1.00) | 0.87 (0.81–0.94)^***^ |
|  | Bupropion | 14,203 | 458 | 110,474 | 4.1 | 0.87 (0.79–0.97)^**^ | 0.87 (0.79–0.96)^**^ | 0.87 (0.79–0.96)^**^ | 0.85 (0.77–0.95)^**^ |
|  | Trazodone | 67,548 | 3,282 | 580,751 | 5.7 | 1.15 (1.09–1.21)^***^ | 1.00 (0.95–1.05) | 0.96 (0.91–1.01) | 0.90 (0.85–0.95)^***^ |
|  | Vortioxetine | 7,697 | 57 | 41,852 | 1.4 | 0.29 (0.22–0.37)^***^ | 0.27 (0.21–0.35)^***^ | 0.27 (0.21–0.35)^***^ | 0.27 (0.21–0.35)^***^ |

DEP, depression; SSRI, selective serotonin reuptake inhibitors; SNRI, serotonin-norepinephrine reuptake inhibitor; TCA, tricyclic antidepressant; MAOI, monoamine oxidase inhibitor

**^a^**Adjusted for age (years) and sex

**^b^**Adjusted for age (years), sex, and Charlson comorbidity Index

**^c^**Adjusted for age (years), sex, Charlson comorbidity index, income, disability, personality disorders, antipsychotic use, benzodiazepine use, stimulant use, mood stabilizer use, and zolpidem use

^*^*P* <0.05, ^**^*P* <0.01, ^***^*P* <0.001

**Supplementary Table 5. Hazard ratios and 95% confidence intervals of antidepressant combinations on type 2 diabetes compared to healthy controls**

|  | **Subjects**  **(n)** | **Events (n)** | **Follow-up duration (person-year)** | **Incidence rate**  **(per 1000 person-years)** | **Hazard ratio (95% Confidence interval)** | | | |
| --- | --- | --- | --- | --- | --- | --- | --- | --- |
|  |  |  |  |  | **Crude** | **Model 1^a^** | **Model 2^b^** | **Model 3^c^** |
| Healthy controls | 276,048 | 24,577 | 3,954,318 | 6.2 | 1 (Ref.) | 1 (Ref.) | 1 (Ref.) | 1 (Ref.) |
| SSRI only | 70,108 | 2,117 | 455,491 | 4.6 | 0.85 (0.81–0.89)^***^ | 1.07 (1.02–1.12)^**^ | 1.02 (0.97–1.07) | 0.86 (0.81–0.90)^***^ |
| TCA only | 48,754 | 5,098 | 439,207 | 11.6 | 2.04 (1.98–2.11)^***^ | 1.61 (1.56–1.66)^***^ | 1.41 (1.37–1.46)^***^ | 1.25 (1.20–1.30)^***^ |
| SSRI+TCA | 41,358 | 2,275 | 413,002 | 5.5 | 0.97 (0.93–1.01) | 0.89 (0.85–0.93)^***^ | 0.77 (0.74–0.81)^***^ | 0.66 (0.63–0.70)^***^ |
| Other combinations | 115,828 | 5,370 | 975,963 | 5.5 | 0.97 (0.95–1.00) | 0.85 (0.82–0.87)^***^ | 0.73 (0.71–0.75)^***^ | 0.60 (0.58–0.63)^***^ |

SSRI, selective serotonin reuptake inhibitors; TCA, tricyclic antidepressant

**^a^**Adjusted for age (years) and sex

**^b^**Adjusted for age (years), sex, and Charlson comorbidity Index

**^c^**Adjusted for age (years), sex, Charlson comorbidity index, income, disability, personality disorders, antipsychotic use, benzodiazepine use, stimulant use, mood stabilizer use, and zolpidem use

^*^*P* <0.05, ^**^*P* <0.01, ^***^*P* <0.001

**Supplementary Table 6. Hazard ratios and 95% confidence intervals of antidepressant combinations on type 2 diabetes compared to DEP without antidepressants group.**

|  | **Subjects**  **(n)** | **Events (n)** | **Follow-up duration (person-year)** | **Incidence rate**  **(per 1000 person-years)** | **Hazard ratio (95% Confidence interval)** | | | |
| --- | --- | --- | --- | --- | --- | --- | --- | --- |
|  |  |  |  |  | **Crude** | **Model 1^a^** | **Model 2^b^** | **Model 3^c^** |
| DEP without antidepressants | 79,119 | 2,898 | 603,168 | 4.8 | 1 (Ref.) | 1 (Ref.) | 1 (Ref.) | 1 (Ref.) |
| SSRI only | 70,108 | 2,117 | 455,491 | 4.6 | 1.01 (0.95–1.06) | 1.19 (1.12–1.26)^***^ | 1.17 (1.11–1.24)^***^ | 1.14 (1.07–1.20)^***^ |
| TCA only | 48,754 | 5,098 | 439,207 | 11.6 | 2.37 (2.26–2.48)^***^ | 1.88 (1.80–1.97)^***^ | 1.74 (1.66–1.82)^***^ | 1.75 (1.67–1.84)^***^ |
| SSRI+TCA | 41,358 | 2,275 | 413,002 | 5.5 | 1.12 (1.06–1.18)^***^ | 1.01 (0.96–1.07) | 0.92 (0.87–0.97)^**^ | 0.91 (0.86–0.96)^***^ |
| Other combinations | 115,828 | 5,370 | 975,963 | 5.5 | 1.13 (1.08–1.18)^***^ | 0.97 (0.93–1.02) | 0.88 (0.84–0.92)^***^ | 0.83 (0.79–0.87)^***^ |

DEP, depression; SSRI, selective serotonin reuptake inhibitors; TCA, tricyclic antidepressant

**^a^**Adjusted for age (years) and sex

**^b^**Adjusted for age (years), sex, and Charlson comorbidity Index

**^c^**Adjusted for age (years), sex, Charlson comorbidity index, income, disability, personality disorders, antipsychotic use, benzodiazepine use, stimulant use, mood stabilizer use, and zolpidem use

^*^*P* <0.05, ^**^*P* <0.01, ^***^*P* <0.001

**Supplementary Table 7. Hazard ratios and 95% confidence intervals on type 2 diabetes among the depression patients with diagnostic codes including symptom severity**

|  |  | **Subjects**  **(N)** | **Events (N)** | **Follow-up duration (person-year)** | **Incidence rate**  **(Per 1000 person-years)** | **Hazard ratio**  **(95% Confidence interval)** | | | | |
| --- | --- | --- | --- | --- | --- | --- | --- | --- | --- | --- |
|  |  |  |  |  |  | **Crude** | **Model 1^a^** | **Model 2^b^** | **Model 3^c^** | **Model 4^d^** |
| Group | |  |  |  |  |  |  |  |  |  |
|  | Healthy controls | 276,048 | 24,577 | 3,954,318 | 6.2 | 1 (Ref.) | 1 (Ref.) | 1 (Ref.) | 1 (Ref.) | 1 (Ref.) |
|  | DEP with antidepressants^e^ | 144,538 | 6,599 | 1,150,379 | 5.7 | 1.03 (1.00–1.05) | 1.02 (0.99–1.04) | 0.92 (0.89–0.95)^***^ | 0.83 (0.79–0.86)^***^ | 0.81 (0.77–0.85)^***^ |
|  | DEP without antidepressants^f^ | 38,777 | 1,298 | 293,745 | 4.4 | 0.79 (0.75–0.84)^***^ | 0.85 (0.81–0.90)^***^ | 0.82 (0.78–0.87)^***^ | 0.75 (0.71–0.80)^***^ | 0.74 (0.69–0.79)^***^ |
| Symptom severity | |  |  |  |  |  |  |  |  |  |
|  | Mild | 86,093 | 3,804 | 682,709 | 5.6 | 1 (Ref.) |  |  |  | 1 (Ref.) |
|  | Moderate | 72,925 | 3,018 | 573,398 | 5.3 | 0.95 (0.90–0.99)^*^ |  |  |  | 1.05 (1.00–1.10)^*^ |
|  | Severe | 24,297 | 1,075 | 188,017 | 5.7 | 1.03 (0.96–1.10) |  |  |  | 1.03 (0.96–1.11) |

DEP = depression.

**^a^**Adjusted for age (years) and sex.

**^b^**Adjusted for age (years), sex, and Charlson comorbidity Index.

**^c^**Adjusted for age (years), sex, Charlson comorbidity index, income, disability, personality disorders, antipsychotic use, benzodiazepine use, stimulant use, mood stabilizer use, and zolpidem use.

**^d^**Adjusted for age (years), sex, Charlson comorbidity index, income, disability, personality disorders, antipsychotic use, benzodiazepine use, stimulant use, mood stabilizer use, zolpidem use, and symptom severity

^e^Subjects who had been diagnosed with depression and prescribed antidepressants.

^f^Subjects who had been diagnosed with depression but not prescribed antidepressants.

^*^*P* <0.05, ^**^*P* <0.01, ^***^*P* <0.001
